# Seasonal variation of blood pressure in children

**DOI:** 10.1007/s00467-020-04823-w

**Published:** 2020-11-19

**Authors:** Niels Ziegelasch, Mandy Vogel, Werner Siekmeyer, Heiko Billing, Ingo Dähnert, Wieland Kiess

**Affiliations:** 1 Hospital for Children and Adolescents, University of Leipzig, Liebigstraße 20a, 04103 Leipzig, Germany; 2 LIFE Leipzig Research Centre for Civilization Diseases, University of Leipzig, 04103 Leipzig, Germany; 3 Pediatric Cardiology, Leipzig Heart Center, 04289 Leipzig, Germany; 4 Centre of Paediatric Research (CPL), University of Leipzig, 04103 Leipzig, Germany

**Keywords:** Seasonal variation, Blood pressure, Children, Temperature, Climate, Pediatrics

## Abstract

Seasonal blood pressure (BP) variation is mostly found between the summer and winter months. Guidelines for diagnosis and treatment of hypertension in children have not considered this variation until recently. This review aims to present an overview of seasonal BP variation in childhood along with potential underlying pathophysiological mechanisms and long-term implications as well as conclusions for future studies. In pediatric cohorts, seven studies investigated seasonal changes in BP. These changes amount to 3.4–5.9 mmHg (or 0.5–1.5 mmHg per − 1 °C difference in environmental temperature) in systolic BP with a peak in fall or winter. Potential mechanisms and mediators of seasonal BP variation include sympathetic activation of the nervous system with an increase of urinary and plasma norepinephrine levels in the winter season. Additionally, the physical activity among children and adolescents was inversely correlated with BP levels. Temperature sensitivity of BP and pediatric BP levels predict future systolic BP and target-organ damage. Therefore, cardiovascular events may even be long-term complications of seasonal BP variation in pediatric hypertensive patients. Overall, these data strongly suggest an important effect of ambient temperature on BP in children. Additional studies in pediatric cohorts are needed to define how best to incorporate such variation into clinical practice.

## Introduction

Seasonal blood pressure (BP) variation is an omnipresent occurrence in the world [1, 2]. The BP differences are mostly found between the summer and winter months. Even in tropical regions, BP varies with outdoor temperature [2]. In 2017, the “Clinical Practice Guideline for Screening and Management of High Blood Pressure in Children and Adolescents” updated the “Fourth Report on the Diagnosis, Evaluation, and Treatment of High Blood Pressure in Children and Adolescents” from 2004, including 30 key action statements and 27 additional recommendations on diagnosis and treatment. New definitions of elevated BP and hypertension were proposed [3, 4]. However, none of these consensus documents and guidelines considered seasonal changes of BP. Nevertheless, medical scientists recommend the consideration of potential seasonal influences in hypertensive as well as normal- and high-risk patients in clinical practice [5].

This review aims to present an overview of seasonal BP variation in childhood along with current recommendations for diagnosis and management of elevated BP in children. Furthermore, potential underlying pathophysiological mechanisms are discussed. In consideration of long-term complications described in adults, we derive directions for future studies in pediatric subjects.

## Seasonal blood pressure variation in children

Most studies on seasonal BP variation collected data in adults. To date, only seven studies have investigated the seasonal variation of BP in children. These are summarized in Table 1.

**Table 1:** Overview of previous studies on seasonal variation in blood pressure in children

	*n* (obs.)	Age (years)	SBPv (mmHg)	DBPv (mmHg)	Additional cohort information
Miersch et al. 2013 [2]	6714	3–21	4.5	2.4	Primarily healthy, Germany
Polat et al. 2006 [6]	547	0–15	5.9	3.6	Primarily healthy, Turkey
Jenner et al. 1987 [7]	1037	9	0.5–0.7/− 1 °C	0.5–0.7/−1 °C	Primarily healthy, Australia
Levinson et al. 1985 [8]	4086	5–10	2.8–4.0	no variation	School children, USA
De Swiet et al.1984 [9]	491	4–5	1.5/− 1 °C	NA	Primarily healthy, Great Britain
Narang et al. 2018 [10]	14,957	5–15	Elevated BP* 29.4% (winter) vs. 18.7% (summer)		Primarily healthy, India
Nika et al.2019 [11]	2832	6–18	Elevated BP* 5.5% (spring) vs. 2.5% (fall)		School children, Greece

BP blood pressure, DBPv diastolic blood pressure variation, NA not assessed, n (obs.) number of observations, SBPv systolic blood pressure variation. SBPv and DBPv represent the difference of blood pressure mean of the winter minus that of the summer. Some studies only appointed the difference as a slope depending on temperature change as indicated by “/− 1 °C”. Elevated BP* was defined as proposed by the “Fourth Report on the Diagnosis, Evaluation, and Treatment of High BP in Children and Adolescents” (Narang et al.) and the “European Society of Hypertension” (Nika et al.)

Miersch et al. in 2013 evaluated the data of 6714 BP measurements in 3 - to 21-year-old healthy and sick children in Leipzig, Germany. Data were collected through routine medical check-ups and monitored in the CrescNet—a screening system for anthropometric development. The mean systolic BP increased by 4.45 mmHg from summer to winter, and diastolic BP increased by 2.42 mmHg. The increase was found even in participants with BP levels in the hypertensive range. Besides, the systolic BP showed a significant negative correlation to outdoor temperature and height, whereas it was positively associated with weight [2].

In 2006, Polat et al. described a similarly significant effect of seasonal changes on both systolic and diastolic BP in 547 primarily healthy children in Manisa, Turkey. Systolic BP was significantly higher in winter (101.2 ± 8.9 mmHg compared to 95.3 ± 15.2 mmHg in summer; *p* < 0.05) as was diastolic BP (67.1 ± 7.3 mmHg vs. 63.5 ± 8.3 in summer; *p* < 0.05). Furthermore, Polat et al. investigated a potential influence of urinary specific gravity and urinary erythrocyte number as indicators of hydration status. No significant change was found in urinary specific gravity or erythrocyte number (*p* > 0.05). Changes in BP were not significant in various age groups (infants, toddlers, school children) probably due to an inadequate number of children in that age group [6].

Older studies include an Australian cohort with 1037 nine-year-old children showing an increase of systolic BP levels during the winter of 0.5–0.7 mmHg per − 1 °C difference in environmental temperature when compared to the summer. The ambient temperature of the day of examination was used for statistical calculations [7]. Furthermore, a US-American study in 1985 included data of 4086 schoolchildren aged 5–10 years screened for seasonal variation of BP. Systolic BP levels were significantly higher in spring (108.8 and 107.4 mmHg) than in fall (105.4 and 106.2 mmHg) and winter (104.8 and 104.6 mmHg for boys and girls, respectively), whereas no summer data were collected because of summer break [8]. Elevated BP values in spring were discussed to reflect faster growth of children in spring or lower room temperature when compared to the winter, where heating systems are used. In British 4- to 5-year-old children, this difference in systolic BP levels amounted to 1.5 mmHg per − 1 °C (*n* = 491 in winter, *n* = 361 in summer) [9].

One Indian and one Greek study compared the prevalence of BP values in the hypertensive range in different seasons. In the Indian study from 2018, Narang et al. screened 14,957 primarily healthy schoolchildren aged 5–15 years in predominantly rural sites for hypertension, as defined by the “Fourth Report on the Diagnosis, Evaluation, and Treatment of High BP in Children and Adolescents” [4, 10]. Elevated systolic BP was diagnosed in 13.6%, diastolic in 15.3%, and both systolic and diastolic in 5.9% of all subjects tested. Overall, hypertension was found more frequently during winter (29.4% of all measurements) compared to summer (18.7%). This effect of seasonal variation remained significant in multivariate analyses. Interestingly, a higher prevalence of hypertension found in northern locations compared to southern areas in India was postulated to be the result of a lower humidity and perspiration and therefore lower loss of sodium [10]. Noteworthy, the term “hypertension” as used by Narang et al. is not applicable and misleading, as BP was only measured on one occasion. For the diagnosis of hypertension, the current guideline demands BP measurements on at least three occasions [3, 4].

A Greek study published in 2019 investigated 2832 6- to 18-year-old children. 3.7% of all subjects showed elevated BP, and 0.9% had BP values in the hypertensive range, as defined by the European Society of Hypertension [11, 12]. The prevalence was highest in spring (5.5%) and winter (5%) and significantly different from that in autumn (2.5%, *p* < 0.05). No significant difference was found for the summer with a prevalence of 4%. Worth mentioning is that the study took place in Kastoria, in the Northern part of Greece, with cold spring temperatures being lower than autumn ones. Other seasonally varying observations of this study included milder sleep problems occurring with hot weather and less physical activity, but increasing workload with exams during the winter months [11].

Overall, the studies report significant seasonal changes of 3.4–5.9 mmHg or 0.5–1.5 mmHg per − 1 °C difference in environmental temperature in systolic BP with higher values in fall or winter [2, 6–9]. Similarly, diastolic BP increased by 2.4–3.6 mmHg or 0.5–0.7 mmHg/−1 °C [2, 6, 7]. Only the American study reported no significant seasonal variation in diastolic BP. A possible explanation may be ethnic differences in this cohort that were shown to affect diastolic BP [8]. The prevalence of BP in the hypertensive range increased by 50% and 57% from warm to cold seasons in the Indian and Greek studies, respectively [10, 11]. The power of most studies is fairly strong with significant numbers of subjects, especially in the studies from India, Germany, the USA, Greece, and Australia [2, 7, 8, 10, 11]. In general, the participants received three BP measurements after resting for 5 min using sphygmomanometers and a properly sized cuff. The mean of all three measurements was used for statistical analyses, with the exception of Nika et al. in Greece using only the last two measurements [11]. Narang et al. in India applied only one BP measurement repeating the assessment only in case of high BP [10]. Finally, Miersch et al. retrieved the data over the CrescNet screening system used by different pediatric clinics. The data collection followed standardized procedures performed by trained medical staff, but details regarding the assessment could not be retraced for every clinic [2].

## Possible pathophysiological mechanisms

During winter, weather conditions may change physiological and biochemical conditions in the human body. Physiological stress and psychological stress, along with sympathetic activation of the nervous system, have been suggested to increase BP levels in colder surroundings [2, 13, 14]. Supportive findings for these associations include studies in children and adults, showing an increase of urinary as well as plasma norepinephrine in the winter, especially in patients with essential hypertension [13, 15, 16].

Additionally, fibrinogen was shown to increase and lead to hypercoagulability in adults during winter months simultaneously to the sympathetic activation [2, 13]. Consequently, Polat et al. analyzed a possible association of the hydration status on seasonal BP variation in 547 primarily healthy children. However, no significant changes in urinary specific gravity, temperature, or humidity levels during different seasons were found [6].

Miersch et al. discuss that weight, heart rate, parameters of lipometabolism, and C-reactive protein (CRP) also have a seasonal pattern, with an increase during winter [2]. CRP and fibrinogen are both inflammation markers suggesting an association of inflammatory activity and BP levels that has not been investigated so far to our knowledge.

A few studies in adult subjects analyzed the potential effect of BMI on seasonal BP variation. A Chinese study of 506,673 primarily healthy subjects showed that BP variation was more distinct in people with lower BMI and higher age [17]. Such inverse correlations were also found by Miquel et al. in Spain and by Woodhouse et al. in an English study. The underlying mechanism remains unclear [18, 19].

In contrast, Youn et al. found no correlation of BMI with seasonal BP changes. However, the pulse wave velocity as an indicator of arterial stiffness was associated with seasonal BP differences in elderly patients with essential hypertension [20]. Nevertheless, arterial stiffness is a phenomenon that cannot explain seasonal changes in BP levels in children.

In another study, seasonality was described quantitatively as the change in daylight hours, proposing an association of seasonal BP changes with the duration of daylight. In 1897 adult patients with hypertension, daylight hours were positively related to nighttime BP, whereas a negative correlation was found towards morning BP surge [21]. On the contrary, Miersch et al. discuss that a potential influence of levels of vitamin D seems unlikely as changes in BP due to changing ambient temperature were shown to be even stronger in tropical countries [2].

Finally, seasonal variation in physical activity among children and adolescents may contribute to seasonal changes in BP. Carson et al. reviewed 35 studies, including children and adolescents between the ages of 2 and 19 years. Overall, seasonal variation in physical activity among the subjects investigated was evident in 29 studies. Neither the region, physical activity measure, nor the study design influenced or mediated this association, but the findings were inconsistent across different age categories [22]. Shephard and Aoyagi reviewed annual physical activity rhythms in children and adults and confirmed an increase in physical activity from winter to spring/summer as described by Carson et al. In children, this increase amounts to + 6.2%. Pedometers and accelerometers even count 6–32% higher during the summer compared to the winter [23]. Additionally, the body height increases relatively faster in spring and fall, possibly explaining lower BP levels in the adapting cardiovascular system [8, 23]. On the other hand, in studies with Black, Hispanic, and especially obese students, a weight gain was evident not only during the winter but also in summer. However, considering lower physical activity during summer vacation, study results are often non-significant and inconsistent [23].

## Potential clinical implications

Seasonal variation in BP may result in insufficient antihypertensive treatment and therefore long-term complications due to hypertension. Given the lack of specific studies on clinical outcomes of seasonal BP variation in children, it is necessary to examine what is known from adult studies.

In adults, a higher risk for cardiovascular events and deaths during winter season is known and associated with hypertension [23–26]. Prospective studies found that the risk for and prevalence of cardiovascular diseases is increased in the cold season with death occurring thrice as often when compared to the summer [27, 28]. An analysis of data from 19 countries of both hemispheres on 54 million deaths was able to confirm seasonal patterns of deaths due to cardiovascular diseases with a peak in winter in both hemispheres. As expected, due to lower annual variation of outdoor temperature in countries close to the equator, those patterns were considerably lower [29]. An increase of mortality during winter was also associated with lower living-room temperatures due to missing indoor heating systems as well as unstable, cloudy weather conditions [30–32]. Further complications of seasonal BP variation include subarachnoid and intracerebral hemorrhage. The prevalence of subarachnoid and intracerebral hemorrhage was higher in the cold season and associated with a decreasing ambient temperature [33–35].

A follow-up study in 169,000 adults in Great Britain found a positive correlation of temperature sensitivity of BP with a higher follow-up systolic BP and even with a higher mortality [36, 37]. Thus, a repeated routine BP monitoring during different seasons may already identify high-risk patients that may benefit from regular follow-up investigations and—where applicable—from suitable treatment. Furthermore, these study results suggest a linkage of seasonally varying BP in temperature-sensitive children and adolescents with cardiovascular events during the later life period. Pediatric elevated BP was shown to be a strong predictor of adult BP levels and future target-organ damage [38, 39]. However, studies directly linking seasonal variation in BP of pediatric patients with adult cardiovascular diseases as long-term complications do not exist until today.

## Summary and future directions

Studies in large pediatric cohorts report significant seasonal changes in BP [2, 7, 8, 10, 11]. These changes amount to 3.4–5.9 mmHg or 0.5–1.5 mmHg per − 1 °C difference in environmental temperature in systolic BP with a peak in fall or winter [2, 6–9]. The prevalence of BP in the hypertensive range increased by 50% and 57% from warm to cold seasons in Indian and Greek cohorts, respectively [10, 11]. Potential mechanisms and mediators of seasonal BP variation include sympathetic activation of the nervous system [2, 13, 14], with an increase of urinary as well as plasma norepinephrine in the winter in both children and adults [13, 15, 16]. Additionally, the physical activity among children and adolescents showed a similar, but inverse annual rhythm compared to BP levels [22, 23]. In adults, associations of increased arterial stiffness, fibrinogen levels, and hypercoagulability as well as lower BMI levels with higher BP levels during the winter months were evident [2, 13, 17–20]. Furthermore, potential clinical implications include cardiovascular events such as myocardial infarction, stroke, and hemorrhage [23–35]. Temperature sensitivity of BP and pediatric BP levels predict future systolic BP and target-organ damage. Therefore, cardiovascular events may even be long-term complications of seasonal BP variation in pediatric patients [36–39]. However, studies directly linking seasonal variation in BP in children with adult cardiovascular diseases are still missing.

When reviewing seasonally varying BP values in children, one limitation is the limited number of studies regarding this topic so far. Studies on pathophysiological mechanisms and potential clinical implications have mostly been performed in adult cohorts. Results of studies in adults may not be completely transferable to children. Moreover, more research regarding seasonal BP variation and the potential underlying mechanisms in pediatric cohorts are needed.

This topic is of high relevance. A regular assessment of BP is essential and recommended at least once a year according to Flynn et al., 2017 [3]. However, especially in patients with antihypertensive treatment or an elevated risk for hypertension, the assessment should take place in different seasons to account for seasonal changes. The prevalence for hypertension in children amounts to 4.2 up to 19.2% in different studies [40, 41].

In obese children, hypertension is a more frequent occurrence. The prevalence of obesity was shown to increase from 1988 to 2008 from 17.8% to 21.8%, and severe obesity from 5.7% to 8.8% [40–42]. Body mass index (BMI), waist circumference, and sodium intake were each independently associated with the prevalence of elevated BP [40]. Simultaneously, the incidence of chronic kidney diseases and hypertension in children increased. Potential pathophysiological mechanisms are the activation of the sympathetic nervous system and the renin angiotensin aldosterone system as well as hyperinsulinemia and inflammatory processes [43]. These mechanisms were also discussed earlier as possible pathophysiological mechanisms for seasonal BP variation. Cohorts of pediatric patients with chronic kidney diseases showed elevated levels of BP in 50–52% of all subjects investigated [44]. Therefore, there is no doubt about the importance of accurate and regular assessment of BP levels in children [5, 45].

In adults with antihypertensive treatment, the timely adjustment of medication to seasonal variation induced a decline of cardiovascular events [46]. Macumber et al. and Chen et al. suggest pediatric elevated BP being a strong predictor of adult hypertension and future target-organ damage [38, 39]. Therefore, early BP screening and, if appropriate, intervention and adaptation of therapy to seasonal changes may be as important to avoid long-term complications as in adults. In order to confirm this thesis, more studies on seasonal BP variation in pediatric cohorts are needed.

## Conclusions

Seven studies report significant seasonal changes of BP in children with higher values in the cold season. Potential mediators include physical activity levels and the sympathetic activation of the nervous system with elevated levels of norepinephrine during winter months. With a high prevalence of hypertension in children, further research and reference values for BP respecting seasonal changes are needed. Additional research may enable an appropriate adaptation of antihypertensive treatment to seasonal BP variations in clinical practice if applicable.

## Key summary points

- Existing consensus documents on children’s BP show a high prevalence of hypertension but do not consider seasonal changes.
- In healthy children, systolic BP levels significantly increase in winter compared to summer.
- Potential mediators include the sympathetic activation with elevated norepinephrine levels and hypercoagulability during winter.
- BP levels in children are shown to predict those in adulthood. In adulthood, an association of elevated risk for cardiovascular events in colder months with higher BP levels is already evident.
- Further studies regarding the modulation of antihypertensive medications according to seasonal variation in pediatric BP are needed.

## Multiple-choice questions (answers are provided following the reference list)

1. What is the increased prevalence of hypertension in children according to a study from 1999 to 2008 for boys and girls?
  a 9.2% and 5.8% respectively
  b 15.8% and 8.2% respectively
  c 12.8% and 15.2% respectively
  d 19.2% and 12.6% respectively
  e 29.1% and 22.6% respectively
2. The prevalence of BP in the hypertensive range in children...
  a decreases by 50-75% in the cold season.
  b decreases by 5-17% in the warm season.
  c increases by 15-17% in the warm season.
  d increases by 17-50% in the cold season.
  e increases by 50-57% in the cold season.
3. The systolic BP change between winter and summer in children described by Miersch et al. amounts to approximately:
  a 2-15 mmHg
  b 2-31 mmHg
  c 2.4 mmHg
  d 4.5 mmHg
  e. 15-31 mmHg
4. According to this review, potential mediators of seasonal blood variation in children seem to include…
  a sympathetic activation of the nervous system, elevated levels of norepinephrine and hypercoagulability, but not vitamin D nor hydration status.
  b sympathetic activation of the nervous system, elevated levels of norepinephrine and hydration status, but not vitamin D nor hypercoagulability.
  c vitamin D and hydration status, but not sympathetic activation of the nervous system, elevated levels of norepinephrine nor hypercoagulability.
  d sympathetic activation of the nervous system, elevated levels of norepinephrine, hypercoagulability and hydration status but not vitamin D.
  e. vitamin D and hydration status, but not sympathetic activation of the nervous system, elevated levels of norepinephrine nor hypercoagulability.
5. What are the main suggestions summarized in this review considering seasonal BP variation in children?
  a regular monitoring in healthy children as well as patients treated for hypertension, more research regarding seasonal BP differences
  b monthly BP measurement in patients treated for hypertension, more research regarding seasonal BP differences
  c monthly BP measurement in patients treated for hypertension, more research regarding pathophysiological mechanisms of seasonal BP differences
  d monthly BP measurement in patients treated for hypertension, more research regarding the long-term effects of seasonal BP differences in children
  e triple BP measurement for every adolescent in the routine check-up examination, more research regarding the long-term effects of seasonal BP differences in children
